# Outpatient Waiting Time in Health Services and Teaching Hospitals: A Case Study in Iran

**DOI:** 10.5539/gjhs.v6n1p172

**Published:** 2013-11-10

**Authors:** Rafat Mohebbifar, Edris Hasanpoor, Mohammad Mohseni, Mobin Sokhanvar, Omid Khosravizadeh, Haleh Mousavi Isfahani

**Affiliations:** 1 Department of Health Care Management, School of Health, Qazvin University of Medical Sciences, Qazvin, Iran; 2 School of Public Health, Tehran University of Medical Sciences, Tehran, Iran; 3 Hospital Management Research Center, Iran University of Medical Sciences, Tehran, Iran

**Keywords:** outpatient, quality, queuing theory, FIFO, hospital

## Abstract

**Background:**

One of the most important indexes of the health care quality is patient’s satisfaction and it takes place only when there is a process based on management. One of these processes in the health care organizations is the appropriate management of the waiting time process. The aim of this study is the systematic analyzing of the outpatient waiting time.

**Methods:**

This descriptive cross sectional study conducted in 2011 is an applicable study performed in the educational and health care hospitals of one of the medical universities located in the north west of Iran. Since the distributions of outpatients in all the months were equal, sampling stage was used. 160 outpatients were studied and the data was analyzed by using SPSS software.

**Results:**

Results of the study showed that the waiting time for the outpatients of ophthalmology clinic with an average of 245 minutes for each patient allocated the maximum time among the other clinics for itself. Orthopedic clinic had the minimal waiting time including an average of 77 minutes per patient. The total average waiting time for each patient in the educational hospitals under this study was about 161 minutes.

**Conclusion:**

by applying some models, we can reduce the waiting time especially in the realm of time and space before the admission to the examination room. Utilizing the models including the one before admission, electronic visit systems via internet, a process model, six sigma model, queuing theory model and FIFO model, are the components of the intervention that reduces the outpatient waiting time.

## 1. Introduction

In response to the certain conditions of each period, the health care organizations have observed major changes. These Various changes included the rapid growth of technology costs of the health sector. Increasing complexity of processes along with the increasing competition among the institutions of health care services have changed the opinion of the experts towards the health care systems providing the health services (Aeinparast, Tabibi, Shahanaghi, & Arianezhad, 2009). Nowadays customer care in all the organizations particularly the health care organizations has advanced and progressed and has a specific symbol. One of the comprehensive factors for the patients in the health care organizations is actually the criteria considered for the suitable and desirable treatment which is rapid and suitable. Prompt treatment in hospitals means to minimize the time for getting a health service with an emphasis on the favorable treatment (Dansky & Miles, 1997). All the organizations providing the health services tend to reduce the number of patients admitted in order to reduce the costs and increase the income, in addition providing opportunities for the people has resulted in the increased number of outpatients over the past decade (Eldabi, Irani, & Paul, 2002).

Changes and major challenges in the health sector have led to the changes in the outpatient services showing the appropriate management of these centers (Vissers, 1998). Delays in the access to the medical services are an important issue for the publicly funded health systems (Schachter, Romann, Djurdev, Levin, & Beaulieu, 2013). Effectively managing patient flow in an outpatient unit is a key to achieve operational excellence as well as ensuring clinical quality. It is especially true for an outpatient department in a large hospital as it handles very large volume of patients with a diverse case mix (Mardiah & Basri, 2013). The waiting time for patients is one of the important factors that should be considered in the management and organization of the health care system. Patients waiting time is not the only factor that affects patient satisfaction but it is one of the indexes to evaluate the quality of outpatient services (Sibbel, Urban, & Saam, 2001). One of the most important indexes of the health care quality is patient’s satisfaction and it takes place only when there is a process based on management. One of these processes in the health care organizations is the process of the appropriate management of waiting time (Boudreaux, d'Autremont, Wood, & Jones, 2004).

Outpatient waiting time has two important aspects. One aspect is that the waiting time of the patients referred by the general practitioner who is created from the time of the entry to the hospital for receiving services, creates great costs and can be modified by techniques of reducing the waiting time. The other one is the waiting time from the time of arrival to the hospital till entering to the examination room. The second part of this time is the waiting time which its major part is related to the hospital and comes to the existence in the hospital. And for reducing the waiting time hospitals should approach through basic interventions. Regarding the first domain, policy makers should provide the essential procedures and guidelines, so that the waiting time for health care services is reduced to the minimum.

Clinics outpatient waiting time is the main focus of this research and is considered to be a center for providing the outpatient services. The importance of the outpatient clinic is that it creates the waiting time for patients and this is due to the structural factors and underlying processes of the clinic, such as working process and physical condition of the clinic. The role of management and laborers in the hospital is important for surveying the outpatient waiting time (Bamgboye & Jarallah, 1994). The waiting time of the outpatient in clinics is formed because a patient who seeks a counseling service spends a period of his time in the hospital, instead of working and earning money.

Dansky presented several definitions for the waiting time. One of these definitions refers to the total time that a patient spends to receive a particular service since entering to the hospital till entering the examination room to visit a doctor. Dansky believes that the patient suffering from his own illness and pain should not suffer from other pains (Leddy, Kaldenberg, & Becker, 2003). The main problem related to the outpatient waiting time is obtained from three main factors (Abdullah, 2005):

1) People (consumers).
2) Organizations providing health services (suppliers).
3) Environment.

Nowadays in the hospitals most of the time organizations themselves refer to the second factor which is involved and plays a role in creating waiting time, and there should be greater emphasis on hospitals to reduce the waiting time. In the educational and learning hospitals of universities under this study, time organizations are related to the rate of the entry of outpatients which makes this subject less interesting and it's follow up requires extensive interventions in this field of research. Thus this study aims at the survey of the outpatient waiting time in two educational and learning hospitals affiliated to the University of Ghazvin.

## 2. Methods

This descriptive cross sectional study is an applicable study. The sampling population of the study in this research includes outpatients visiting the educational and learning hospitals affiliated to the University of Ghazvin. In the beginning in order to ensure the equal distribution of patients in different months of the year, the rate of entry and exit of all the patients in all the clinics under the study in the year 2010 were surveyed. Since the distributions of outpatients in all the months were equal, sampling stage was used.

STAGE 1: Random selection of two health clinics from all the clinics presented in each hospital: Of the clinics presented in two hospitals, urology and orthopedic clinic from hospital A and ophthalmology and dermatology clinic from hospital B was selected.

STAGE 2: randomly selected outpatients were used to collect the sample society from the following sample size formula.


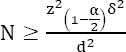


Within a time range of 4 minutes and accuracy of 99% a standard deviation of 18.98 minutes (pilot sample) for 160 persons was obtained. 160 outpatients under the study were divided among four clinics (ophthalmology, dermatology, urology and orthopedics). To ensure the accuracy of the data, the number of outpatients was divided equally during the two weeks and the waiting time was measured on the basis of minutes/second in the presence of a researcher by chronometer.

For gathering the information check lists, we used the researcher certified by the opinion of five teachers. The method for collecting the information was through the presence in hospital and by the observations and interviews performed by the researchers. The data was analyzed by using descriptive mean test, standard deviation, variance and by the software SPSS. The specific variables for measuring the waiting times were evaluated as follows:

1) Waiting time before admission.
2) Waiting time in the reception part.
3) Waiting time in funds part.
4) Waiting time before examination.

## 3. Results

After analyzing the data, the following results were obtained. Out of a total of 160 outpatients, 53% were males and 47% were females. 6/97% of the study population lived in the rural areas of Ghazvin. 11/62% of the residents lived near the suburbs of Qazvin and the remaining 81.41% lived in the Qazvin city itself. The education level of the study population is shown in the table 1.

**Table 1:** Level of education as per degree

Level of education as per degree	percentage
Illiterate	9.30%
Primary	9.31%
FAQ	15.11%
Diploma	27.90%
Associate degree	7.71%
Bachelor	25.58%
Above bachelor	5.09%

**Figure 1:**
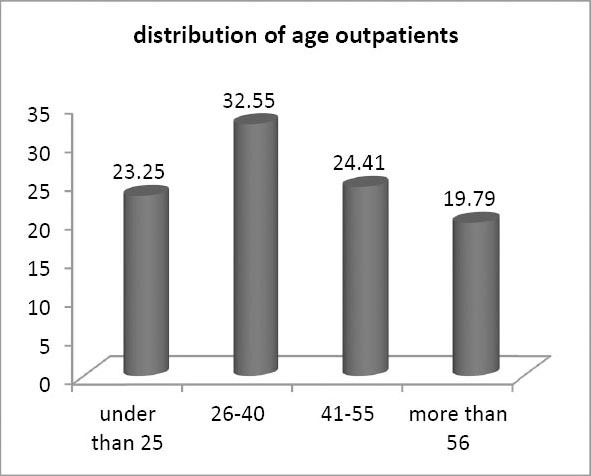
Age distribution of the outpatient under study

In this study, the waiting time for the outpatients of ophthalmology clinic with an average of 245 minutes for each patient allocated the maximum time for itself. This time was 130 minutes (52.84%) in relation to the distance between the cash department and the examination room. 113 minutes (45/93%) belongs to the waiting time of the clinics before the admission. Altogether these two parts created a waiting time of 243 minutes equivalent to 98/78%.

But the minimum waiting time belongs to the orthopedic clinic with an average of 77 minutes allocating to each patient. Out of 77 minutes of waiting time about 65 minutes (83/33%) of it belongs to the distance between the cash department and the examination room. Approximately 10 minutes (12/82%) of the waiting time belongs to the pre admission stage.

The dermatology clinic comparing with the clinics under the study allocated most of the waiting time for the outpatient. The time duration of 216 minutes was allocated for the outpatients and about 86 minutes (46/08%) were related to the pre admission and 59/44% of it belonged to the distance between the cash department and the examination room.

The waiting time of urology clinic was also less than others and the duration of time for each outpatient was 81 minutes. Most of this time belongs to the distance between the cash department and the examination room (85/18%). According to the analytical data obtained from this study, it can be said that most of the waiting time is related to the distance between the cash department and the examination room. 100 minutes from the total waiting time of the outpatient (approximately 161 minutes) that is 61/72% apparently makes this subject clear. However a lot of time was wasted in the department prior to the admission which is about 59 minutes. These two departments accounted for 98/13% of the total waiting time. Therefore the main problem of these two variables under the study can be identified. 81/4% of the epidemiologic study population lived in the city of Qazvin and approximately 66/28% of them had an education level higher than diploma and this strengthens the interventions and models to reduce the waiting time. Table 2 shows the mean total waiting time for each patient and the duration of visits for selected clinics.

**Table 2:** Average of waiting time and visiting time for every outpatient

Duration in minutes Clinic	Total waiting time	Visit time
	Mean±SD	Mean±SD
Dermatology clinic	216±32	4±0.8
Ophthalmology clinic	245±29.8	6±1
Urology clinic	81±41.6	4±0.7
Orthopedic clinic	77±43.4	6±0.8

Table 3 shows the average of waiting time and visit time for every outpatient in the selected hospital. This waiting time is unto four domains: pre admission, admission, fund, and before the visit room. Finally, the total of the waiting time has been showed for every outpatient.

**Table 3:** Duration of the waiting time for hospitals under study

Duration in minutes	Mean ±SD
Pre admission	59±20.1
Reception department	1±0.2
Funds department	1±0.2
Before examination	100±30.5
The total waiting time	161±36.7

## 4. Discussion

On the basis of the entry of the outpatients in the year of this study and equality and acceptance of outpatients in each clinic during all the months, these samples can give us enough information. Our sampling population is enough educated and spends a lot of waiting time in the queues of the hospital to get the health services. The more the educational level and the awareness of the community is, the better health services patients will get, and they can take the advantage of the new acceptance system. However patients only should not be convicted for excessive waiting time. This phenomenon has been converted into a social problem in the developed and developing countries (Chand, Moskowitz, Norris, Shade, & Willis, 2009).

In our study, the waiting time for the outpatients of ophthalmology clinic with an average of 245 minutes for each patient, allocating maximum time for itself was 130 minutes (52.84%) in relation to the distance between the cash department and the examination room. 113 minutes (45/59%) belongs to the waiting time of the clinics before the admission. Altogether these two parts created a waiting time of 243 minutes equivalent to 98/78%. Waiting time in the reception department and the funds department as compared with these two sectors had a significant difference. It should be stated that the waiting time in two reception departments and the funds is acceptable and close to the standards of the hospital (Aeinparast et al., 2009). In the two other departments serious strategies should be considered. But about the minimum waiting time, it can be said that in orthopedic clinic with the lowest waiting time an average of 77 minutes was allocated for per patient. Out of 77 minutes of the waiting time about 65 minutes (83/33%) of it belongs to the distance between the cash department and the examination room. Approximately 10 minutes (12/82%) of the waiting time belongs to the pre admission stage. The dermatology clinic also as compared with the clinics under the study, allocated most of the waiting time for the outpatient. The time duration of 216 minutes was allocated for the outpatients and about 86 minutes (46/08%) were related to the pre admission time and 59/44% of it belongs to the distance between the cash department and the examination room. The waiting time of urology clinic was also less than others and the duration of time for each outpatient was 81 minutes. Most of this time belongs to the distance between the cash department and the examination room (85/18%). According to the analytical data obtained from this study, it can be said that most of the waiting time was related to the distance between the cash department and the examination room. 100 minutes from the total waiting time of the outpatient (approximately 161 minutes) that is 61/72% apparently makes this subject clear. However a lot of time was wasted in the department prior to admission, which is about 59 minutes. These two departments accounted for 98/13% of the total waiting time. Therefore the main problem of these two variables in the study can be identified. 81/4% of the epidemiologic sampling population lived in the city of Qazvin and approximately 66/28% of them had an education level higher than diploma and this strengthens the interventions and models to reduce the waiting time. In the United Kingdom, because of the national health insurance and a wide range of health services this waiting time is less than other countries. Rodney, the statistical consultant in the health research center of England, has estimated the waiting time of outpatients in his studies to be 35 minutes. However this waiting time was allocated for the distance between the admissions of the patient and the examination room for getting the consultation. Rodney considers the referral system effective for reducing the waiting time of the outpatient and effective for the increased duration of visits. He believes that if unnecessary referral of patients is avoided by the family physician, waiting time of patients is reduced and physicians can spend more time for their patients (Jones, 2000).

These results were obtained from the study that Chen performed with the cooperation of medical information management center along with the management of the research centers of Japan in the university hospitals of Guangzhou and China about the visiting time of the physician and waiting time of the outpatient. In this study the visiting time for each patient in the morning was about 42 minutes. This time the duration was less than the afternoon which reached to 25 minutes. And overall average of 33 minutes time duration for each patient is spent by the physicians visit. However the number of patient’s admission in the morning and afternoon shift were less and limited also. Chen relates it to the academic hospitals and believes that in these hospitals the waiting time of patients and the time duration of visit and consultation is more; Which is affected by the education and learning of assistants. In this hospital, the community reservation system is used for the admission of the patients. In this system few days before the arrival of the patient and the patient’s admission, processes of registration are performed. Reservation system has been created from the industrial and commercial systems and the basic foundation of this system has been established in the financial and sales market (Chen et al., 2010).

In a study conducted by Irshad Rahim in the hospitals of Dhaka Bangladesh in the year 2007, total waiting time for each outpatient from the registration process to the entry of the examination room was estimated to be 66 minutes. He stated at the end that this duration of time is very high and the managers of the hospital should undertake appropriate measures to reduce it (Rahim, Rahman, Talukder, & Anwar, 2007).

In the hospitals under the study, the main problem can be identified by the process model. Increased and long waiting time in these hospitals is related to the time before the admission and to the distance between the cash department and the examination room. Approximately 98/13% of the total waiting time for these two departments shows the importance of these two centers. Intervention in these centers of waiting time can lead to the improvement of other sections such as funds department and the reception.

In 1995, Shaw presented in his model that the waiting time for receiving the health services is affected by the demand. He linked these factors to the health care demands of the consumer and stated that if the waiting time of the patient for receiving the health services is too high, The patient can move from the main center to the other private sectors for getting services and even the traditional medicine (Asefzadeh, 2008). Factors within and outside the hospital are involved in generating the waiting time. They are identified by using some techniques such as network diagrams and fish bone diagrams (Lim & Manaes, 2001). In the clinic of hospital B, although the patients would wait for long time before admission, but all of them would be accepted in a short time. It seems that false claim is seen in these patients. These factors need to open people’s attitude towards engineering. However acceptance of increased number of patients could not be ignored. According to the network analysis, four factors were involved in the long waiting time of the patients (Sharif & Sukeri, 2003).

1) Human Resources: lack of human resources and professional workers in the hospitals such as physicians, licensed nurses and other professional staff.
2) Equipment and facilities: the type of information system used in hospitals is one of the major factors. Most of the hospitals use the same old admission systems on the same day.
3) Patients: patients themselves and their attitudes are involved in the increased waiting time.
4) Registration process.

Despite the long waiting time for outpatients, their visiting time was extremely low. Visiting time of 5 minutes in learning and educational hospitals created a place for challenging the subject. Although the expertise opinion and insight of different specialists in making correct diagnosis is different, but the patient has the right to get a typical expert advice for a standard duration of time (Helbig, Helbig, Knecht, Kahla-Witzsch, & Gstöttner, 2007).

Waiting time leads to the worsening of the patient’s disease which needs consultation with the doctor. The best quality services in health care organizations are the right of every individual. One of the most important tools to improve the quality of health care services is to reduce the long waiting time (Matthews et al., 1991). One of the interventional methods for detecting long waiting time and its resolution is performed by six sigma approaches.

During the last 5 years or so, many leading healthcare institutions have implemented Lean and Six Sigma methodologies with remarkable results in terms of reducing cycle time (Gijo, Antony, Hernandez, & Scaria, 2013). Mohamed Hanafi Abdullah in his study in Malaysia utilized this model to reduce the waiting time. His innovative approach led to the relationship between this model and the approach of the Pareto rule (Abdullah, 2005). And after identification of the axis for creating long waiting time, 80% of the problems related to the long waiting time as the result of the following three factors:

1) Registration process.
2) Shortage of physician
3) Shortage of professional staff

### 4.1 Types of Models to Reduce the Outpatient Waiting Time

#### 4.1.1 Admission Model before the Acceptance of the Patient

According to this model the patient should not have the waiting time before the admission. In order to implement this method, a reservation system that would function in two ways was used. One of them was admission through telephone. In this method, the patient would contact the hospital by phone and would provide the employees of reception with the basic information. Another reservation system was through the computer. Patients could enter their basic information in the reception department of the outpatient through the internet site of the hospitals. In this case, an officer in the charge of the reception would organize the information. By implementation of this model, when the patients require a consultation, they would wait for less than a minute in the reception department. This time duration for registering the information and ensuring the accuracy of the data was filled up by the patients themselves (Lee & Yoo, 1996). This model can be used in the learning hospitals (university hospitals) with diverse expertise to gain great success. Developing countries with the launch of advertising campaign can draw people’s attention for using these systems.

#### 4.1.2 Electronic Visit System

The best present system to reduce the waiting time is the electronic visit through the web network. This model is obtained from the doctor’s consultant and patient’s relationship. Most of these models are used in hospitals and private clinics. The costs overhead of the clinics have also been reduced by using these systems. The biggest limitation of this system is the lack of patient physician and consultant relationship in the emotional level. Moreover, these systems are mostly used in specialties such as psychiatry. In this case, the physician and patient can meet with each other, if necessary. The experts promote use of this system for family physician and the specialist relationship. This system is expanded and works on three zones, the patient, family physician and specialist. This system works quiet well in the developing countries and particularly in the developed countries with more advanced and extensive medical information systems and new applications (Taylor, 2006).

#### 4.1.3 Queuing Theory

This model suggests that in order to reduce the queues of waiting outpatient, there should be an increase in the patient admission booth and an increase in the number of examination rooms. This model is used when the outpatient number is too high. In addition, sufficient human and physical resources should be present. This model has more functionality in learning hospitals affiliated with the university because of the human funds (Helbig et al., 2007).

#### 4.1.4 Process Model

Perhaps the best model for identifying and solving the problems of outpatient waiting time in the hospitals under the study is the use of a process model. This model presents that the process should be examined to identify the problem (Willcox et al., 2007). We found that from the outpatient's reception till the entry into the examination room, most of the waiting time is related to the distance between the cash department and the examination room and the patient’s reception (98/13%). This model suggests that the factors having more effect on this process should be considered more than other factors and the long term processes should be reduced or eliminated. For example according to this model processes before acceptance of the patient which takes the long waiting time (36/41%) should be eliminated. For this purpose acceptance model can be used before the admission. For example physician specialists can begin sooner visiting their patients, or they can start later or even patients can be admitted later by accepting the patients through simulation model. It should be noted that the faculty physicians in learning hospitals are mostly specialist and should begin counseling immediately after the admission of the first patient. This will lead to the greater utilization of time and will devote more time to the patient during the examination.

#### 4.1.5 FIFO Model (first in first out)

This technique is known as the queue priority and is also compared with the queue of getting bread. It’s not been too long that this model is used. The FIFO technique is a new approach used for the analysis of the waiting time of the outpatients and its reduction. This technique is described as a queue priority. One who is admitted first will be the last person who gets out of the admission queue. This process can be applied to the cash department and the examination room also. Finally the patient admitted first will be the last person exiting from the examination room. This technique is mostly considered for the emergency department as priority plays an important role there. However this technique is used in the entire process from pre admission till the exit of the patient from the examination room (Ozturk, Di Mascolo, Espinouse, & Gouin, 2010).

The specific Time duration for each patient’s visit by doctor is short (5 minutes). This time duration should be at least 15 minutes for each patient. Despite the different diagnostic approach of a physician specialist for the patient and the amount of fee paid by the patient, it is the patient's right to get examined for a longer time. Perhaps one of the reasons behind the frequent visits of patient to the clinic is the shorter time duration which is specified for them. In addition to it, this duration of time can indirectly increase the waiting time of the patients. It can be hypothesized for the visiting time duration and the waiting time of outpatients for the future research.

Time, like human resources and money, is not renewable and it’s not reversible also. It’s the right of the people to make the best use of their time. If the hospitals environment is so pleasant that the waiting time of patient’s is converted from pain to a state of joy and comfort, then it can compensated for the prices paid by the patient (Helbig et al., 2007).

## 5. Conclusions

According to the present study, The Overall total average waiting time for each patient was about 161 minutes. 161 minutes waiting time is a lot for each outpatient in the hospitals. The models mentioned above can be effective for reducing the waiting time of the outpatient. The adoption of the model before admission and the process model has more functionality in the hospitals under this study. Since the waiting time of the patient was related to pre admission and the distance between the cash department and the examination room, utilization of these models is considered to be a great priority. But in the shorter period of doctor’s visit, there should be much supervision and control in the health care organizations and they should suit the adopted policies.

## References

[ref1] Abdullah MH (2005). Study on Outpatients’ Waiting Time in Hospital University Kebangsaan Malaysia (HUKM) Through the Six Sigma Approach1. The Journal of the Department of Statistics, Malaysia.

[ref2] Aeinparast A, Tabibi JT, Shahanaghi K, Arianezhad MB (2009). Estimating outpatient waiting time: a simulation approach. Payesh.

[ref3] Asefzadeh S (2008). Health economic.

[ref4] Bamgboye EA, Jarallah JS (1994). Long-waiting outpatients: target audience for health education. Patient education and counseling.

[ref5] Boudreaux ED, d’Autremont S, Wood K, Jones GN (2004). Predictors of emergency department patient satisfaction: stability over 17 months. Academic emergency medicine.

[ref6] Chand S, Moskowitz H, Norris JB, Shade S, Willis DR (2009). Improving patient flow at an outpatient clinic: study of sources of variability and improvement factors. Health Care Management Science.

[ref7] Chen B, Li E-d, Yamawuchi K, Kato K, Naganawa S, Miao W-j (2010). Impact of adjustment measures on reducing outpatient waiting time in a community hospital: application of a computer simulation. Chinese Medical Journal (English Edition).

[ref8] Dansky KH, Miles J (1997). Patient satisfaction with ambulatory healthcare services: waiting time and filling time. Hospital & health services administration.

[ref9] Eldabi T, Irani Z, Paul RJ (2002). A proposed approach for modelling health-care systems for understanding. Journal of Management in Medicine.

[ref10] Gijo E, Antony J, Hernandez J, Scaria J (2013). Reducing Patient Waiting Time in a Pathology Department Using the Six Sigma Methodology. Leadership in Health Services.

[ref11] Helbig M, Helbig S, Knecht R, Kahla-Witzsch H, Gstöttner W (2007). Quality management: reduced waiting time and enhanced efficiency in a university ear, nose, and throat outpatient department. HNO.

[ref12] Jones R (2000). Outpatient appointments. Feeling a bit peaky. Health Serv J.

[ref13] Leddy KM, Kaldenberg DO, Becker BW (2003). Timeliness in ambulatory care treatment. An examination of patient satisfaction and wait times in medical practices and outpatient test and treatment facilities. J Ambul Care Manage.

[ref14] Lee YJ, Yoo DH (1996). A study on model for decreasing an outpatient waiting time. Journal of Korean Society of Medical Informatics.

[ref15] Lim W, Manaes M (2001). Benchmarking waiting times for clinic services, Kuala Lumpur Hospital. Journal of Quality Improvement.

[ref16] Mardiah FP, Basri MH (2013). The Analysis of Appointment System to Reduce Outpatient Waiting Time at Indonesia's Public Hospital. Human Resource Management Research.

[ref17] Matthews F, Probert C, Battcock T, Frisby S, Chandar M, Mayberry J (1991). Are we wasting time in out-patients departments?. Public Health.

[ref18] Ozturk O, Di Mascolo M, Espinouse M.-L, Gouin A (2010). Minimizing the sum of job completion times for washing operations in hospital sterilization services.

[ref19] Rahim R, Rahman M, Talukder AM, Anwar KH (2007). waiting time of the patients at medical outpatient department of Dhaka medical college hospital. J. med. sci. res.

[ref20] Schachter ME, Romann A, Djurdev O, Levin A, Beaulieu M (2013). The British Columbia Nephrologists’ Access Study (BCNAS) – a prospective, health services interventional study to develop waiting time benchmarks and reduce wait times for out-patient nephrology consultations. BMC nephrology.

[ref21] Sharif JHM, Sukeri S (2003). Study on waiting time at the paediatric dental clinic, Kuala Lumpur Hospital. Journal of Quality Improvement.

[ref22] Sibbel R, Urban C, Saam N (2001). Agent-based modeling and simulation for hospital management. Cooperative agents: applications in the social sciences.

[ref23] Taylor A (2006). electronic visit in East Kent Hospitals NHS. journal of medical sciences.

[ref24] Vissers JH (1998). Health care management modelling: a process perspective. Health Care Management Science.

[ref25] Willcox S, Seddon M, Dunn S, Edwards RT, Pearse J, Tu JV (2007). Measuring and reducing waiting times: a cross-national comparison of strategies. Health Affairs.

